# Applying Case-Based Reasoning to Tactical Cognitive Sensor Networks for Dynamic Frequency Allocation

**DOI:** 10.3390/s18124294

**Published:** 2018-12-06

**Authors:** Jae Hoon Park, Won Cheol Lee, Joo Pyoung Choi, Jeung Won Choi, Soo Bin Um

**Affiliations:** 1 Department of Information and Telecommunication Engineering, Soongsil University, Seoul 06978, Korea; pjh901118@soongsil.ac.kr; 2 School of Electronic Engineering, Seoul 06978, Korea; pyoung424@soongsil.ac.kr; 3 The 2nd R&D Institute—1st Directorate, Agency for Defense Development, Daejeon 34060, Korea; jwchoi@add.re.kr (J.W.C.); sbum1989@add.re.kr (S.B.U.)

**Keywords:** tactical cognitive radio sensor network, case-based reasoning, cognitive radio engine, channel occupancy probability, military tactical communications

## Abstract

In this paper, a cognitive radio engine platform is proposed for exploiting available frequency channels for a tactical wireless sensor network while aiming to protect incumbent communication devices, known as the primary user (PU), from undesired harmful interference. In the field of tactical communication networks, there is an urgent need to identify available frequencies for opportunistic and dynamic access to channels on which the PU is active. This paper introduces a cognitive engine platform for determining the available channels on the basis of a case-based reasoning technique deployable as a core functionality on a cognitive radio engine to enable dynamic spectrum access (DSA) with high fidelity. To this end, a plausible learning engine to characterize the channel usage pattern is introduced to extract the best channel candidate for the tactical cognitive radio node (TCRN). The performance of the proposed cognitive engine was verified by simulation tests that confirmed the reliability of the functional aspect, which includes the learning engine, as well as the case-based reasoning engine. Moreover, the efficacy of the TCRN with regard to the avoidance of collision with the PU operation, considered the etiquette secondary user (SU), was demonstrated.

## 1. Introduction

**Figure 1:**
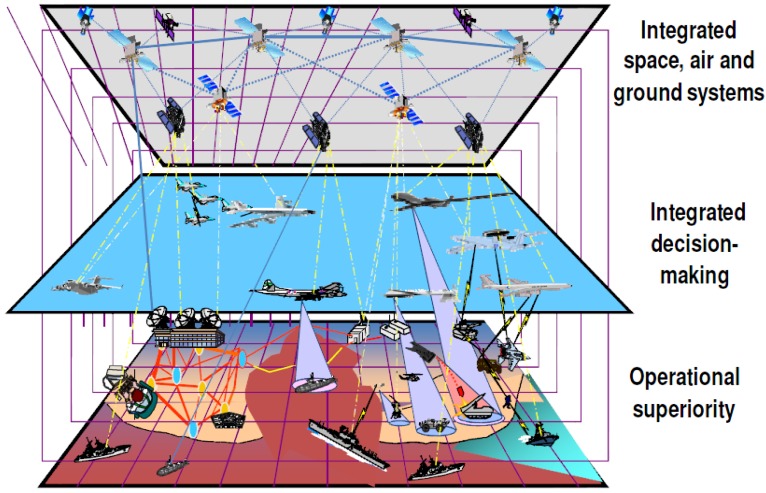
Spectrum utilization environment in the network-centric warfare [6].

The demand for access to additional frequencies is continuously and rapidly increasing for military wireless communications due to the evolution and diversification of tactical weapon systems. In addition, it is gradually becoming more difficult to acquire additional frequencies for military wireless communications because of the increase of commercial-side frequency demand with the advent of 5G and IoT (Internet of Things) [1]. The identification of additional frequency resources for the purpose of military applications, such as tactical sensor networks, is considered to be a major issue of great importance to fulfilling the demand for interoperability while complying with various tactical weapon systems including wireless surveillance equipment, which is one of the usage cases of a wideband sensor network in terms of addressing the C4I perspective (command, control, communication, and computer intelligence) [2]. It has been emphasized in many published reports that the supply of spectrum resources for reliable military weapon system operations is a very urgent matter, considering that military frequency has a term of 10 years [3]. Military operations, which have an evolutional tendency, are transitioning toward network-centric warfare that is based on innovative, advanced, intelligent frequency management schemes [4,5,6]. Therefore, providing operating frequency resources at the right time and to the right place is a primary objective that must be fulfilled, subject to the satisfaction of stable management and quality of service (QoS) requirements. In this context, this paper proposes a cognitive radio engine platform that embeds a learning engine and a case-based reasoning engine. The proposed platform is capable of effective frequency allocation without producing harmful interference, which is a prime concern for the secure coexistence of tactical cognitive radio nodes (TCRNs) in the presence of a primary user (PU) in a military tactical wireless communication environment. Figure 1 shows the future military spectrum management environment to support the network-centric warfare.

There is a relevant study on a cognitive radio (CR) engine that is similar to the one proposed in this paper, which presents a technique for the dynamic exploitation of available channels not occupied by the primary user for the CR node user by observing the channel environment via spectrum sensing [7]. This paper shows the method’s ability to avoid collision with the primary user on the specific channel currently used by the CR node user. However, the exclusion of a learning engine means that if the primary user utilizes frequency hopping (FH), the reliability of the spectrum sensing is naturally lowered because the hopping pattern is not acquired, so the chance of collision between the primary user and the CR node becomes obviously considerable. Moreover, the next-generation (XG) project conducted by the Defense Advanced Research projects Agency (DARPA) for the performance evaluation of the CR engine was carried out as a field test that varied the primary user’s channel utilization [8]. A primary traffic pattern was generated on the basis of mathematical modeling, and the CR-based routing protocol was tested to determine whether it was applicable to an ad hoc network environment [9]. This paper focuses on the application scenarios, design methods, and implementation of the CR engine. The CR engine platform proposed in this paper consists of a cognitive engine, learning engine, reasoning engine, and optimization engine. We also designed a function and operation method for the individual engines and then conducted a performance analysis through simulation.

This paper introduces a PU traffic model, which describes the activity pattern of a PU channel occupancy and proposes a case-based reasoning method deployable to tactical sensor networks. In addition to characterizing the PU traffic status, the paper also proposes a learning engine that is employed to identify available candidate channels. Section 2 discusses the current state of development of cognitive radio engine platforms, whereas Section 3 proposes the structure of the cognitive radio engine platform, together with its functionality. In addition to the PU traffic model, the quantification of the occupancy probability and the case-based reasoning technique with its applications are also reviewed. Section 4 reports the simulation results, which verify the superior performance of the proposed case-based reasoning technique coupled with a learning engine. Section 5 presents concluding remarks.

## 2. Current Cognitive Radio Research and Development for Military Usage

Currently, the United States uses Link-16 tactical communications networks that are equipped with direct sequence spread spectrum (DSSS) and frequency hopping (FH) for jamming the enemy’s communications and defending against attacks [10,11]. Link-16 is based on a non-IP-type fixed hardware platform. Due to the limited spectrum resource, it is necessary to define the number of users and the frequency hopping pattern beforehand. Because of Link-16’s style of predefined fixed wireless resource allocation, it is difficult to use in a modern weapon system’ wireless network, which has an increased need for high-capacity data transmission.

DARPA in the United States developed its XG program to realize a cognitive engine whose major role is the acquisition of the surrounding radio resources via spectrum sensing equipped with a cognitive radio engine, which consists of a policy engine (composed of the learning and reasoning engines) that helps new entrants of tactical wireless networks to acquire appropriate frequencies while avoiding interference with preexisting incumbent users [10]. The cognitive radio engine platform is based on the policy engine developed by DARPA XG, and it is broadly composed of “policy language”, “on-node policy components”, and “off-node policy components” [12]. One of the “on-node policy components” is the “Policy Database”, which stores the policy reasoning data and policy priorities generated by the “Policy Conformance Reasoner”, including the policy data for using cognitive radio nodes [13]. The learning engine introduced in the XG program stores policy strategies on the existing PU-occupied channels, and the reasoning engine arranges the priority of these channel for data transmission by the learning engine. The policy strategy, expressed in data format, is renewed by comparing the PU’s occupied channel data acquired at the current time point with a reference—the previously-stored occupied channel history—and then, the renewed data are sent to the learning engine. The proposed learning engine can be seen as the “Policy Database” entity in DARPA’s policy engine, and the reasoning engine can be considered to play the role of the “Policy Conformance Reasoner”.

**Figure 2:**
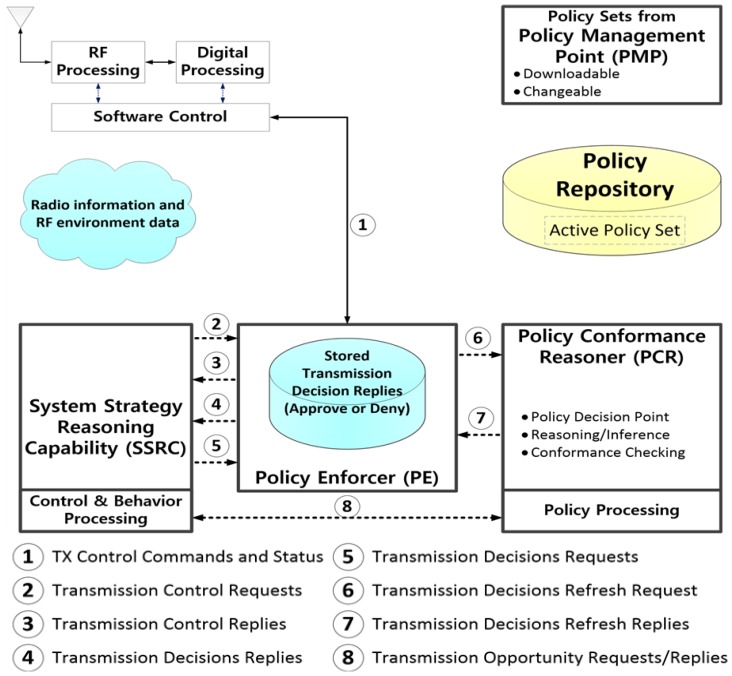
IEEE P1900.5-based cognitive radio engine processing diagram.

The IEEE P1900.5 standard presents the functional definition and requirements relevant to a cognitive radio engine and policy language deployable to policy-based wireless systems [14,15,16]. Table 1 shows the main components and functions that comprise the cognitive radio engine proposed by the IEEE P1900.5 standard. Figure 2 shows the data transfer flow and the related interface between the cognitive radio engine components described in Table 1.

## 3. Cognitive Radio Engine Platform Usage Scenario

### 3.1. Structure and Roles of the Proposed Cognitive Radio Engine

**Figure 3:**
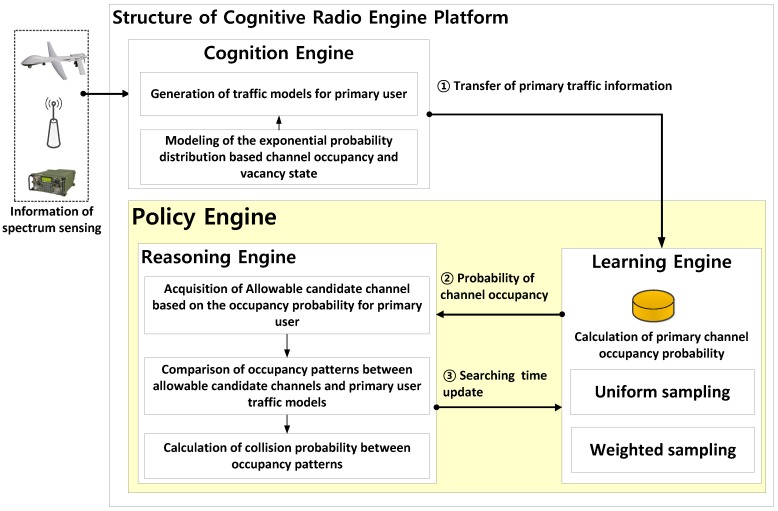
Proposed cognitive radio engine structure.

The proposed cognitive radio engine platform requires a separate step to calculate the probability of PU channel occupancy. This allows for the estimation of the PU channel usage pattern by using a functionality of the cognition engine equipped with spectrum sensing. In this paper, the spectrum sensing observations of the PU channel occupancy pattern are assumed to be perfect, and the pattern obtained via actual spectrum sensing is considered complete. In general, the cognitive engine is composed of four types of engine modules consisting of the cognition engine, the learning engine, the reasoning engine, and the optimization engine. Among those, this paper only deals with two specific engines, i.e., the learning engine, as well as the reasoning engine, introducing their functionalities and capabilities. For given PU traffic patterns associated with every channel, in order to inspect their performances, we assume that the cognition engine works perfect so that the sensed information indicating whether the PU exists or not is correct. The reason why this assumption is made is attributed to calculating the collision probability as a tool of assessment for the performance of the reasoning engine. Therefore, along the step for the PU traffic generation, at first, the distinctive parameters $\lambda_{\alpha }^{n}$ and $\lambda_{\beta }^{n}$ are specified, which yield probabilities $P_{off}^{n}$ and $P_{on}^{n}$ for each channel; with regard to these, the PU traffic is artificially generated in the sequel. Since the sensing process is accomplished without invoking any detection error, the resulting PU traffic represented in two-state binary fashion for each channel is exactly the same as the result of the cognition engine. Furthermore, this PU traffic pattern is subsequently utilized for estimating the PU occupancy probability by applying the individual methods of sampling approaches with varying either the number of samples or the sampling interval. In addition, the PU state duration time is presumed to be random following an exponential distribution, and the occupancy patterns corresponding to every channel are independent. Figure 3 depicts the entire structure of the proposed cognitive radio engine platform.

In Figure 3, one of the major roles assigned to the learning engine is the quantification of the PU occupancy probability, which is obtained from observations in the temporal domain for every channel to be inspected. This information is delivered by the cognition engine. The process of quantifying the PU occupancy probability associated with a certain channel is performed by sampling the PU’s channel usage patterns during a fixed time slot and then calculating the channel occupancy. In this regard, this study applied either uniform sampling or weighted sampling, the details of which are explained later. Furthermore, one of the major roles of the reasoning engine is the exploitation of a group of available candidate channels on behalf of the TCRN for dynamic access to the spectrum in which the PU is possibly active. As the side information from consolidating the set of available channels, the PU traffic pattern constructed by the PU occupancy probability is transferred from the learning engine.

The process for choosing the available candidate channels is implemented by appropriately arranging the channels in ascending order, which creates a series that specifies the PU occupancy probability per channel, where the top-ranked channel is the first priority candidate to be used. Next, all the ranks calculated by every sampling technique considered in this paper for all of the inspected channels are used to form a composite ranking. Ultimately, a group of available channel candidates for the cognitive radio node is selected based on guaranteeing, to the greatest extent possible, a minimum collision probability. The methods of assigning ranks considered in this study are classified into the rank-sum, based on the sum of the ranks of the occupancy probability values of each channel, and the prob-sum, which selects a group of available channel candidates referring to the sum of the occupancy probabilities of each channel. To verify the performance of the reasoning engine, the number of collisions with an existing PU, whose traffic pattern was a priori prescribed, is counted with the assumption that the TCRN uses the channel assigned by the reasoning engine. In addition, the number of samples and reasoning period were considered to be important steering parameters to achieve the optimal available channel candidates.

### 3.2. Modeling Primary User Traffic in a Cognition Engine

The application scenarios of the CR engine were designed with a focus on developing a simulated environment, predicting wireless channels that are not used by the primary user, and actively selecting the channels that have a low probability of PU occupancy. In the simulation scenario, the PU were assigned to individual channels, and the number of licensed channels was assumed to be equal to the total number of primary users. It was also assumed that the PU is licensed to the station in the assigned frequency band and does not leave the frequency band. At this time, it is noted that the use of a channel by a PU is considered to be independent for each individual channel, and channel use can change over time. In this environment, for the selective use of the vacant channel, i.e., one not used by the primary user, the CR node needs the technology to detect the vacant channel and the ability to selectively use it.

**Figure 4:**
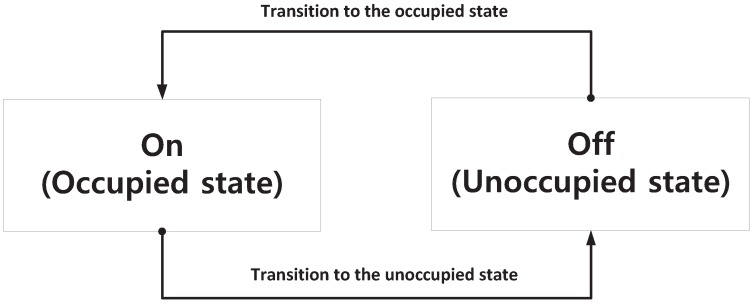
Conceptual diagram of changes in PU channel occupancy states.

The procedure for generating the PU traffic model is addressed in this subsection using an appropriate statistical distribution of the PU’s channel occupancy pattern using data from spectrum sensing. Firstly, it was assumed that the PU channel usage patterns for each channel are independent and that the PU channel occupancy data acquired are perfect. Figure 4 is a conceptual diagram representing the process of switching the PU state between ON and OFF.

Figure 4 reflects the PU channel state transitions, and Equation (1) is the formulation for the probability of a PU being in an OFF state relative to all observed channels [18]:

(1) $$P_{off}^{n}=\frac{\lambda_{\alpha }^{n}}{\lambda_{\alpha }^{n}+\lambda_{\beta }^{n}.}$$

In addition, Equations (2) and (3) define the exponential probability distribution functions, in which the mean value $\lambda_{\alpha }^{n}$ of the random variable is the duration time interval of the PU OFF state, whereas the mean value $\lambda_{\beta }^{n}$ is the duration time interval of the PU ON state:

(2) $$f_{\alpha ,n}\left(x\right)=\left\{\begin{array}{cc} \lambda_{\alpha }^{n}e^{-\lambda_{\alpha }^{n}x} & \;x\geq 0\\ 0 & \;x<0 \end{array}\right.\;,$$

(3) $$f_{\beta ,n}\left(x\right)=\left\{\begin{array}{cc} \lambda_{\beta }^{n}e^{-\lambda_{\beta }^{n}x} & \;x\geq 0\\ 0 & \;x<0 \end{array}\right.\;.$$

**Figure 5:**
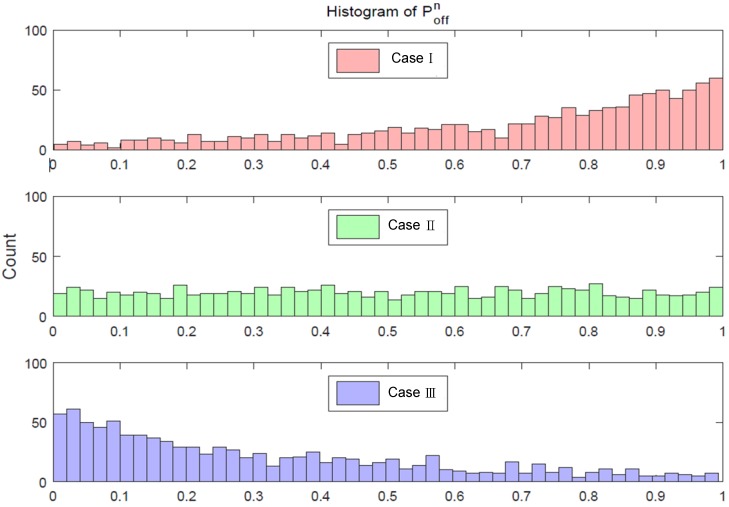
Off-state probability histogram of the primary user.

Here, it should be noted that the PU’s channel usage can be dynamically adjusted by modifying the $\lambda_{\alpha }^{n}$ and $\lambda_{\beta }^{n}$ values. Specifically, Equation (3) is used to calculate the average value of $P_{off}^{n}$ versus *N*, the total number of channels to be observed. In this paper, we assume that all the frequency channels are completely utilized by distinct PUs having their own traffic patterns. Here, each traffic pattern is solely dependent on the fixed constant $P_{off}^{n}$, which is not a random variable, specified by mean values of exponentially-distributed time durations relevant to the PU OFF state, as well as the PU ON state. Since the number of PUs is equivalent to that of frequency channels, many PU traffic patterns are required to be generated in order to conduct the simulation. For the sake of the artificial generation of traffic patterns, which are mutually distinct, it is necessary to make an arbitrary choice of the probability $P_{off}^{n}$ in the range of [0,1] for each channel. Therefore, to assess the performance of the proposed channel allocation scheme based on the case-based reasoning, the collision probability should be measured as a major indicator considering how much the proposed method is appropriately working. Moreover, in the simulation, the case-based reasoning is carrying out the functionality of characterizing the PU traffic in a certain confined interval of reasoning time with regarding to the histogram pattern of $P_{off}^{n}$ resulting in the opportunity of assigning channels for CR nodes. Figure 5 shows the distribution in the form of a histogram of $P_{off}^{n}$, designated as the probability of PU being OFF, where the total number of channels *N* is 1000.

As shown in Equations (2) and (3), $\lambda_{\alpha }^{n}$ and $\lambda_{\beta }^{n}$ are not random variables, but constant values, whose values are distinctive depending on the channel. More specifically, those values are nothing but the average of the exponentially-distributed random duration of time associated with the PU OFF state and ON state, respectively. To create the artificial simulated environment, in which all the channels experience distinctive PU traffic patterns, the corresponding mean values $\lambda_{\alpha }^{n}$ and $\lambda_{\beta }^{n}$ are specified differently on purpose. Along the way of achieving the result as in Figure 5, a priori, 1000 pairs of $\lambda_{\alpha }^{n}$ and $\lambda_{\beta }^{n}$ are generated for every channel to be assigned, and subsequently, the histogram of $P_{off}^{n}$ is generated. In Figure 5, Case I, Case II, and Case III reflect the three distinct PU traffic situations, where Case I indicates that most of the value of $\lambda_{\alpha }^{n}$ is larger than that of $\lambda_{\beta }^{n}$, Case III reflects the opposite case, and Case II deals with the case that most of the value of $\lambda_{\alpha }^{n}$ is almost similar to that of $\lambda_{\beta }^{n}$.

As can be seen in the preliminary simulation results in Figure 5, for Case I, there are many channels in the OFF state, so that the frequency of PU channel occupancy is low. Thus, there would be a high opportunity for the usage of the cognitive radio node. On the other hand, for Case III, the updated histogram reflecting channel occupancy probabilities is slightly skewed to the left, which means that the PU traffic is heavy, so the opportunity for usage becomes low. After generating the value of $P_{off}^{n}$ for the aforementioned OFF state of the PU channel in terms of $\lambda_{\alpha }^{n}$ and $\lambda_{\beta }^{n}$, the next step is to define the instant of time at which the PU activity state changed. Equation (4) determines whether or not the PU state changes for a particular unit of time, designated by the time slot *t* at channel index *n*. After the $P_{off}^{n}$ value is set, a random variable based on the “Russian roulette” method is created to model the state change of the channel that satisfies the idle probability for the channel. At this time, the occupied state value for the corresponding channel is set to zero or one by comparing the uniform distribution random variable *q* and the $P_{off}^{n}$ value. Once the random variable *q* is chosen in the range of 0<q<1, the PU state can be specified as the following:

(4) $$S_{n}\left(t\right)=\left\{\begin{array}{cc} 0 & \;q\leq P_{off}^{n}\\ 1 & \;q>P_{off}^{n} \end{array}\right.\;.$$

**Figure 6:**
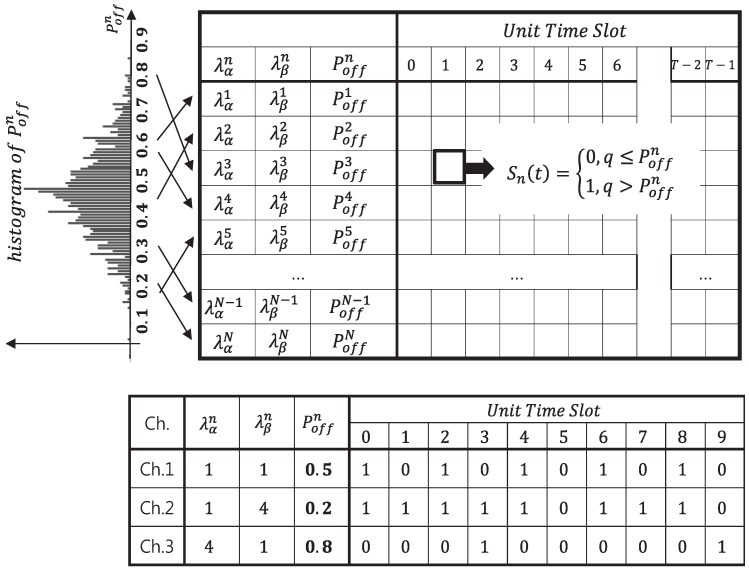
State transitions for occupied and unoccupied states of each channel modeled as zero and one, respectively.

Figure 6 shows a conceptual diagram for the generation of the PU traffic in which the PU channel in the occupied or unoccupied state at each time slot is designated as zero or one, respectively, as defined in Equation (4).

For example, Figure 6 shows the modeling of a state transition associated with three channels. Among them, for the case of Ch.1, $\lambda_{\alpha }^{n}$ and $\lambda_{\beta }^{n}$ are the same such that the “OFF (0)” state and the “ON (1)” state appear at the same ratio, and their mean values are one. Similarly, other kinds of PU traffic models can be generated by assigning PU ON and OFF state patterns through the statistical modeling previously described.

### 3.3. Occupancy Probability Calculation in the Learning Engine

During the execution of the learning engine, the PU occupancy probability with respect to each observed channel can be calculated by counting the time slots for which PU is in the ON state over the entire observation time *T*. It can also be expressed as the exact theoretical formula shown in Equation (5) [18]:

(5) $$P_{on}^{n}=\frac{\lambda_{\beta }^{n}}{\lambda_{\alpha }^{n}+\lambda_{\beta }^{n}}\;.$$

**Figure 7:**
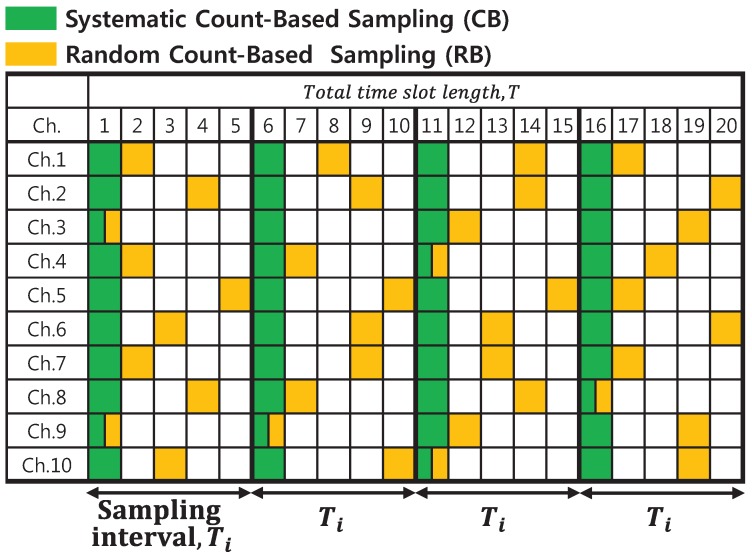
Uniform sampling technique concept and process for the CB and RB methods.

Using the approach for calculating PU occupancy probability making use of empirical observations instead of Equation (5), it is necessary to employ a sampling method for the acquisition of the PU’s channel usage patterns with a high degree of accuracy. In this regard, this study applied uniform sampling and weighted sampling methods. The uniform sampling method initially divides the overall time interval *T* into individual unit time slots with a uniform size and estimates the PU channel occupancy probability from the samples associated with PU traffic. Furthermore, the calculation methods of uniform sampling for PU channel occupancy probability can be divided into systematic count-based sampling (CB) and random count-based sampling (RB), which are distinguishable according to the size of the sampling interval, which is defined as the individual unit time slot [19,20]. Figure 7 depicts the process for uniform sampling for a case in which the mean value of the “OFF” and “ON” intervals is one.

As in Figure 7, the CB method involves acquiring samples at consistently fixed time slots over every sampling interval. On the other hand, the RB method specifies the time slot to be sampled at random over each sampling interval. Consequently, the PU occupancy probability can be calculated by observing the samples that contain the state of the PU traffic. Equation (6) calculates the occupancy probability of the *n*th channel using observed samples obtained by the CB sampling method. Equation (6) can also be applied to the RB sampling method.

(6) $$P_{CB}^{n}=\frac{1}{M}\sum_{m=1}^{M}S_{n}\left(m\right)\;.$$

In Equation (6), *M* indicates the overall number of samples, and $S_{n}\left(t\right)$ represents the status of the PU activity indicator so that zero reads as the ON state and one reads as the OFF state at the *m*th time slot for channel *n*. In this paper, besides the conventional systematic and random count-based sampling methods, their weighted versions are proposed namely as WCB and WRB respectively. More precisely, by applying these approaches, the PU occupancy probability can be estimated by considering the temporal correlation. Therefore, relatively high-valued weights are assigned for recently-acquired samples, whereas older samples are weighted by small values. In this case, the weight can be interpreted by the well-known forgetting factor, and the corresponding weighting process primarily tapers the samples observed during the application of the CB and RB methods. Equation (7) corresponds to the estimated PU occupancy probability via the WCB sampling method [20].

(7) $$P_{WCB}^{n}=\sum_{m=1}^{M}w_{m}S_{n}\left(m\right)\;,$$

(8) $$w_{m}=\frac{1}{K}e^{m},K=\sum_{m=1}^{M}e^{m}\;.$$

With the assistance of Equation (8), the greatest weight is multiplied by the most recent sample indexed by *M*, and the lowest value is multiplied by the oldest sample. Furthermore, *K* in Equation (8) is the normalization factor and is equivalent to the sum of the exponential values. Similar to Equations (7) and (8), WRB-based PU occupancy probability calculations can be performed on randomly-chosen samples. Table 2 summarizes the features of uniform sampling and weighted sampling.

### 3.4. Available Channel Extraction Method via Conducting the Reasoning Engine

#### 3.4.1. Reasoning from the PU Traffic Reference Model

In this paper, we propose a novel channel selection method in which the CR node selects a suitable unoccupied channel, and the channel with the lowest probability of being occupied by the primary user among vacant wireless channels is compared with the previous occupation activity. The CR engine is designed to select the proper candidate channel that is inducing the low level of the collision probability. We also evaluated the performance of the CR engine by comparing the occupancy patterns in a primary user traffic model generated by the mathematical modeling performed by the cognitive engine. In addition, appropriate reasoning and learning parameters, such as the reasoning cycle, sample number, sampling interval, and so on, for satisfying the collision probability requirements of the system were calculated.

Clearly, since the collision probability could not be directly controlled, the proposed method employs the case-based reasoning, which is an indirect way of retrieving appropriate parameters associated with the reference model of PU traffic inducing a low level of collision probability. Here, the reference model plays a major role attributed to giving the CR node a versatile opportunity of selecting relevant parameters. The case-based reasoning is triggered by specifying the feature of the current PU traffic pattern via conducting the shape matching process as a way of comparing the feature relevant to the newly-acquired histogram reflecting the current PU traffic with that stored in the reference model in a clustered and sorted manner. For the next step, the simple extraction process for searching parameters in the reference model is executed. For the purpose of achieving a high degree of precision on the selection of parameters, quite a few arbitrary PU traffic generations should be conducted subject to ensuring the collision probability is as low as possible.

The proposed reasoning method could not perfectly protect the PU from the interference induced by the SU because the candidate channels are only parameters exploited from referring to the PU traffic model. Here, the reference model is constructed by conducting an enormous amount of PU traffic generations followed by executing the deliberate clusterization steps to execute the PU traffic matching. This whole process can be regarded as the learning engine, which can provide adequate information about the reasoning period, the sampling period, and the number of samples for precise traffic matching together with the available channel after the sorting and ranking process proposed in this paper. It is valuable to emphasize that these relevant parameters are exploited by conducting an exhaustive search in the direction of minimizing the collision probability. Assuming that there is no prior knowledge about the channel state and PU activity in this paper, at the instant of starting a new reasoning period, the SU only can transmit the signal over one of the channels suggested by conducting the reasoning process.

This paper considers the case-based reasoning approach to extract a group of available channels for a cognitive radio node operating in a tactical sensor network. For this objective, inspection of the histogram representing the channel usage pattern of a PU is initially conducted using the learning engine. During the second step, the resulting histogram is matched with reference traffic models previously stored in the learning engine; this step is to determine which traffic model is highly similar. During the third step, as the final step in the case-based reasoning process, the group of channels is extracted. To provide some insight into the procedure for matching the PU traffic pattern, Table 3 delivers a few examples that highlight the feature of characterizing the shape of the histogram in terms of the mean, variance, skewness, and kurtosis. There are nine reference traffic models, which can be distinguished from each other by analyzing these intrinsic signatures that determine the shape of the histogram.

In Table 3, the first traffic model in the leftmost column has the lowest mean value, and the mean value gradually increases toward the ninth traffic model. Thus, the first traffic model indicates the situation of low PU activity for every channel, whereas the ninth traffic model corresponds to high PU activity. In this work, the PU traffic models stored up to the previous reasoning period are regarded as reference PU traffic models. The reasoning process determines which traffic model among the reference PU traffic models matches the newly-acquired histogram constructed by the recently-observed samples that are relevant to the PU traffic. In this case, to assess the degree of similarity, the mean and the second, third, and fourth central moments can be utilized to match the traffic models [21,22]. The mean value of $P_{on}^{n}$ is denoted as the central moment associated with the histogram representing the distribution of the frequency of the PU occupancy probability value. The *k*th central moment is defined as in Equation (9),

(9) $$\begin{array}{c} {\hat{\mu }}_{k}=\frac{1}{N}\sum_{n=1}^{N}{\left(P_{on}^{n}-P_{on}^{Avg}\right)}^{k}. \end{array}$$

Furthermore, the relationships introduced in Equation (10) describe how to generate the second moment through the fourth moment, i.e.,

$${\hat{\mu }}_{2}=m_{2}-\mu^{2},\;\;\;\;\;\;\;\;$$

(10) $${\hat{\mu }}_{3}=m_{3}-3\mu_{2}+2\mu^{3},\;\;\;\;\;$$

$${\hat{\mu }}_{4}=m_{4}-4\mu m_{3}-6\mu^{2}m_{2}-3\mu^{4}$$

where $\mu =P_{on}^{Avg}=\frac{1}{N}\sum_{n=1}^{N}P_{on}^{n}$, the second central moment becomes the variance of $P_{on}^{n}$, and the third and fourth central moments are used to achieve the skewness and the kurtosis. With the help of Equation (10), the skewness $\gamma_{1}$ and the kurtosis $\gamma_{2}$ are calculated by Equation (11):

(11) $$\gamma_{1}=\frac{{\hat{\mu }}_{3}}{{\hat{\mu }}_{2}^{3/2}}\;,\;\gamma_{2}=\frac{{\hat{\mu }}_{4}}{{\hat{\mu }}_{2}^{3}}-3\;.$$

The mean, variance, skewness, and kurtosis corresponding to the *i*th traffic model, which is one of *I* models comprising the overall PU reference traffic model structured for the previous reasoning period, are calculated based on recently-acquired samples. For the purpose of assessing the similarity, Equation (12) evaluates the individual errors in the mean, variance, skewness, and kurtosis between the overall reference PU traffic model and the recently-constructed PU traffic model as follows:

(12) $$\begin{array}{c} \varepsilon_{\mu }\left(i\right)=\frac{|\mu^{ref}\left(i\right)-{\hat{\mu }}^{mea}|}{\mu^{ref}\left(i\right)},\;\;\varepsilon_{\mu_{2}}\left(i\right)=\frac{|\mu_{2}^{ref}\left(i\right)-{\hat{\mu }}_{2}^{mea}|}{\mu_{2}^{ref}\left(i\right)},\\ \varepsilon_{\gamma_{1}}\left(i\right)=\frac{|\mu_{\gamma_{1}}^{ref}\left(i\right)-{\hat{\mu }}_{\gamma_{1}}^{mea}|}{\mu_{\gamma_{1}}^{ref}\left(i\right)},\;\;\varepsilon_{\gamma_{2}}\left(i\right)=\frac{|\mu_{\gamma_{2}}^{ref}\left(i\right)-{\hat{\mu }}_{\gamma_{2}}^{mea}|}{\mu_{\gamma_{2}}^{ref}\left(i\right)}\;. \end{array}$$

If a certain index of the traffic model is determined to be an index of the reference, the PU traffic models give the composite error, as specified by Equation (13). It can be concluded that the corresponding two traffic models are coincident, so the verification of the model for newly-incoming PU traffic is completed as follows:

(13) $$\min_{i\in I}\left(\varepsilon_{\mu }\left(i\right)+\varepsilon_{\mu_{2}}\left(i\right)+\varepsilon_{\gamma_{1}}\left(i\right)+\varepsilon_{\gamma_{2}}\left(i\right)\right)\;.$$

By applying an exhaustive search to identify the correct index of the reference PU traffic model, it is subsequently possible to acquire information about the optimal reasoning period and the optimal number of samples that minimize the collision probability, which are also stored in the reasoning database.

#### 3.4.2. Extracting Available Channel Candidates from Reasoning Results

To exploit the group of available channels for allocation to the cognitive radio nodes, a sequence is first arranged of the PU occupancy probabilities in ascending order, and the ranking of all the channels is decided. This study examined the performance related to the PU collision probability by applying individual sampling methods, including uniform sampling methods (CB and RB) and weighted sampling methods (WCB and WRB), together with rank-sum and prob-sum reasoning methods. In this case, collision probability refers to the probability of the event in which the cognitive radio node incorrectly uses a channel that is already occupied by the PU.

To this end, the *j*th channel is ranked as $R_{CB}\left(j\right)$, $R_{RB}\left(j\right)$, $R_{WCB}\left(j\right)$, and $R_{WRB}\left(j\right)$, which result from the action of the cognition engine followed by the learning engine, and *R* is an integer that obeys 1≤R≤N. For example, the rank of channel *j* calculated by executing the CB sampling method is shown in Equation (14) [18]:

(14) $$R_{CB}^{}\left(j\right)=rank\left(P_{CB}^{j}\right)\;.$$

Here, the rank() function is used to decide the ranking of every channel by assessing the occupancy probability, which is calculated and sorted in the learning engine. The range of the rank is shown in Equation (15) and is the same as the overall number of channels based on the following:

(15) $$Range\left\{R_{CB}\left(1\right),\ldots ,R_{CB}\left(N\right)\right\}=N\;.$$

Furthermore, Equation (16) realizes the proposed rank-sum reasoning approach, which aggregates ranks derived from the execution of each sampling method. As a result, based on this method, the channel with the minimum rank sum is regarded as a candidate channel. This paper denotes this approach as the rank-sum reasoning method, with the relevant formulation given as:

(16) $$R_{Rank-sum}\left(j\right)=R_{CB}\left(j\right)+R_{RB}\left(j\right)+R_{WCB}\left(j\right)+R_{WRB}\left(j\right)\;.$$

Besides this, Equation (17) defines the prob-sum reasoning method that specifies the candidate channel by sorting the sum of the occupancy probabilities resulting from each sampling method in ascending order, given by:

(17) $$R_{Prob-sum}\left(j\right)=P_{CB}\left(j\right)+P_{RB}\left(j\right)+P_{WCB}\left(j\right)+P_{WRB}\left(j\right)\;.$$

Similar to the rank-sum reasoning method, the channel with the minimum probability sum is designated as a candidate channel. In the following, Table 4 shows the PU occupancy probabilities represented in percent, which is quantified by adopting the individual sampling method. The rank-sum and prob-sum methods give rise to the composite ranking referred to as CB, RB, WCB, and WRB.

As shown in Table 4, the reasoning method proposed in this paper for determining candidate channels using prob-sum does not select the channel with the lowest PU occupancy probability for a specific sampling approach, but selects a reliable candidate channel, which is the most competitive channel with respect to either the composite ranking or the aggregated occupancy probability. Moreover, another critical point is how many reliable channels are determined to clarify this; a guideline can be recommended based on the PU reference traffic model containing relevant information corresponding to the traffic-matched subject for minimizing the collision probability.

## 4. Simulation

### 4.1. Simulation Scenario

**Figure 8:**
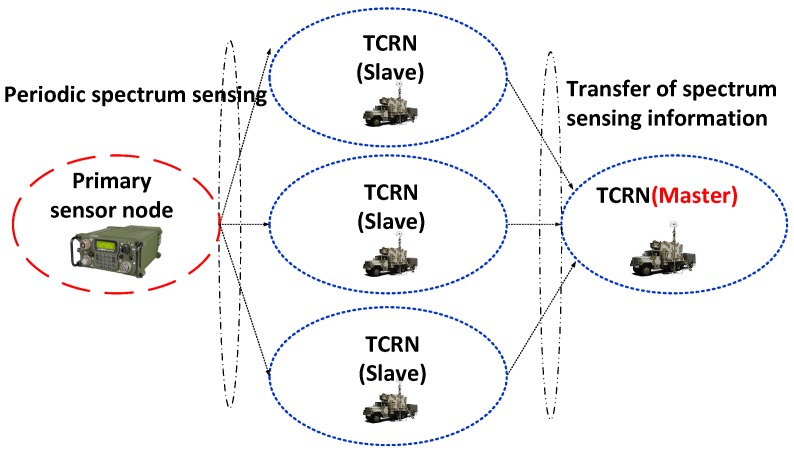
Simulation scenario environment. TCRN, tactical cognitive radio node.

Figure 8 shows a simulation scenario for evaluating the performance of the proposed cognitive engine platform. In Figure 8, there is one PU radio required to be protected from the interference induced by neighboring cognitive radio nodes. In addition, four cognitive radio nodes are deployed in the simulation environment: one plays the role of master, and the others operate in slave mode. The slave-type TCRNs perform spectrum sensing functionality to observe PU activity periodically, which reads the channel occupancy state. Furthermore, every slave-type node transports the acquired sensing information reflecting the channel occupancy state to the master TCRN. Then, the master TCRN makes a decision as to which channels are appropriate so that cognitive radios equipped with the cognitive engine use those channels in a secure manner, without interfering with the PU.

**Figure 9:**
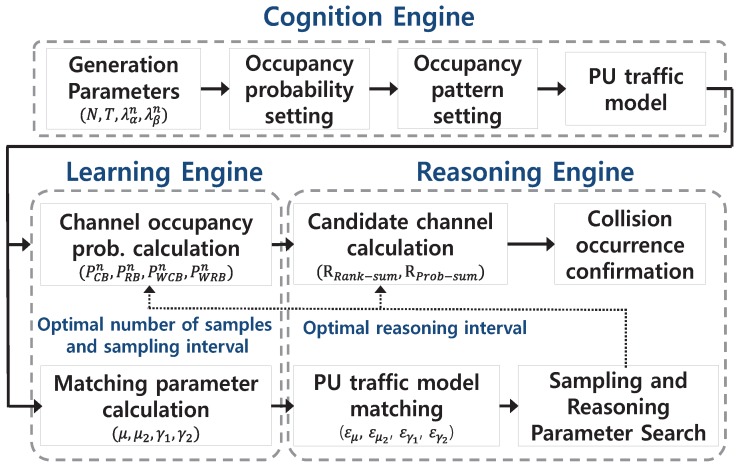
Simulation analysis flowchart.

In the simulation, the PU channel occupancy characteristic acknowledged by the master TCRN with the help of spectrum sensing technology was artificially generated so as to produce a PU traffic model following a specific statistical distribution. Figure 9 shows a diagram of the simulation process.

As shown in Figure 9, the PU traffic model can be characterized based on inspecting a probability distribution having the form of an exponential distribution. In other words, the temporal duration of the ON and OFF states statistically follows an exponential distribution. The learning engine uses the PU traffic model generated in the previous reasoning period to quantify the PU channel occupancy probabilities that correspond to the overall channels based on CB, RB, WCB, and WRB. Finally, the reasoning engine, assisted by the rank-sum and prob-sum methods, gives rise to available candidate channels for the usage of the cognitive radio node. After performing a series of assignments, the probability associated with the a priori PU traffic prescribed for the simulation is calculated in order to verify the efficacy of the proposed cognitive engine.

**Figure 10:**
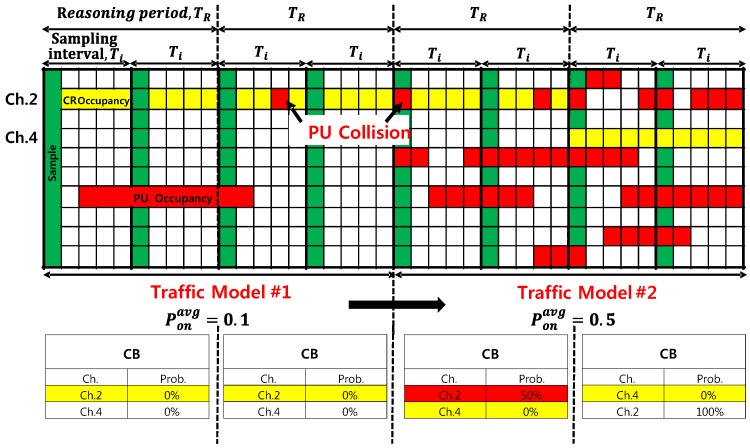
Definition of collision and an example of a periodic operation of the CR engine.

To better describe this process, Figure 10 depicts an example of the consecutive operation of the CR engine. As a result, the candidate channels can be exploited at every reasoning period. The learning engine newly updates the occupancy probabilities obtained by each sampling method and replaces those with the previous ones at the end of each reasoning period if needed. The reasoning engine exploits the available candidate channels for a given TCRN. This process is repeated periodically, and the suitable reasoning period and the number of samples can be achieved as byproducts by matching the recently-acquired traffic pattern with the preceding traffic model, resulting in a similar distribution to the PU occupancy probability.

### 4.2. Simulation Results

**Figure 11:**
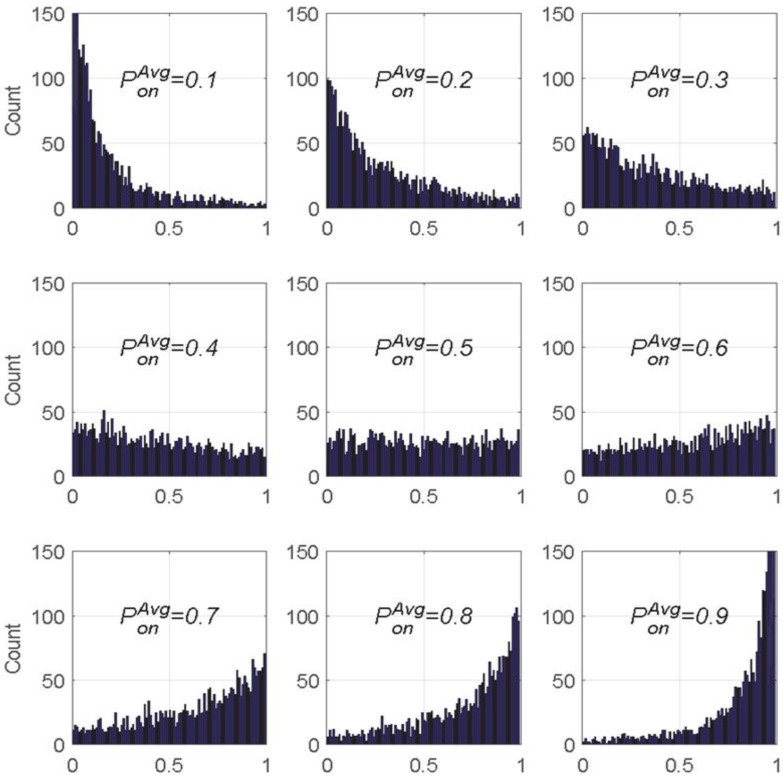
Results of PU traffic model generation based on the exponential probability distribution *M* = 2700).

Figure 11 shows a series of histograms relevant to the PU traffic model created in the learning engine. The histograms present the distribution of the PU occupancy probability. In Figure 11, when the value of $P_{on}^{Avg}$ is 0.1, 0.2, 0.3, or 0.4, a channel’s PU occupancy state probability value $P_{on}^{n}$ is generally found in positions that have small values. When the $P_{on}^{Avg}$ value is 0.5, a histogram is distributed with left-right symmetry. Further, when the $P_{on}^{Avg}$ value is 0.6, 0.7, 0.8, or 0.9, the PU occupancy probability $P_{on}^{n}$ seems to be biased toward positions of large values.

**Figure 12:**
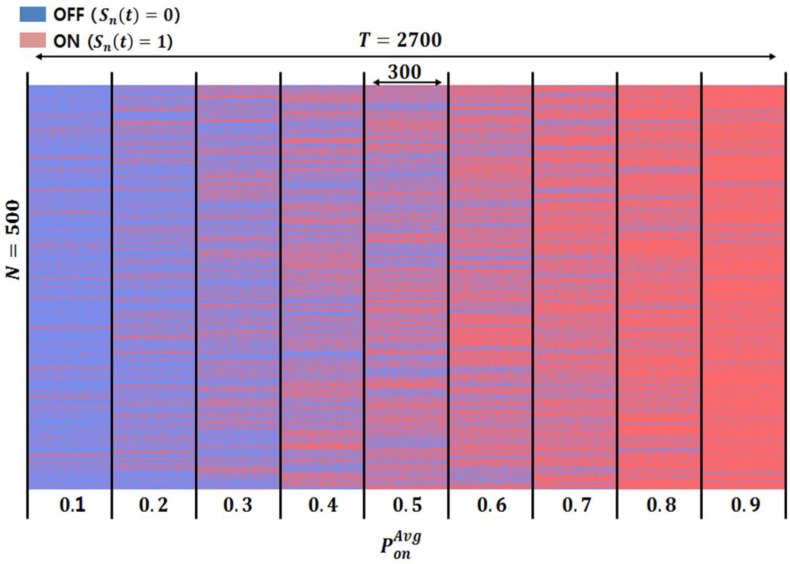
Result of traffic model generation according to the average value of the channel occupancy probability of the PU.

Table 5 shows the parameter type and their corresponding values used to verify the performance of the case-based reasoning method proposed in this paper deployed in the cognitive radio engine platform. Further, Figure 12 shows PU reference traffic models in which the average of the channel occupancy probability $P_{on}^{Avg}$ was enforced to be incremented from 0.1–0.9. As a result, nine PU traffic models are consecutively generated for every time slot interval, which was set to 300 slots per model. The overall time slot interval *T* was set to 2700 slots, and the total number of channels *N* was set to 500. In Figure 12, the OFF state is shown in blue for the channels of the cognitive radio nodes that are available. The ON state shown in red implies that the cognitive radio nodes could not find available channels. Overall, as the value $P_{on}^{Avg}$ (the average probability of the PU channel occupancy) increases, it is evident that the frequency of PU occupancy also increases.

#### 4.2.1. Collision Probability Behavior According to Sampling and Reasoning Methods

Here, the collision probability is similar to a sort of outage probability for the PU. However, it is not exactly the same because the collision probability in our paper is nothing but the result of counting the number of time slots where both PU and SU exist together without considering the values such as the signal-to-interference-plus-noise ratio (SINR) or the interference-to-noise ratio (I/N). Thus, even though there is a collision occurring at a certain time slot, the interference may not be harmful to the PU since the level of SINR or I/N is acceptable because those satisfy a priori prescribed requirements. This could possibly happen when the PU is located far from the SU or the SU is intentionally blocked by the clutter.

**Figure 13:**
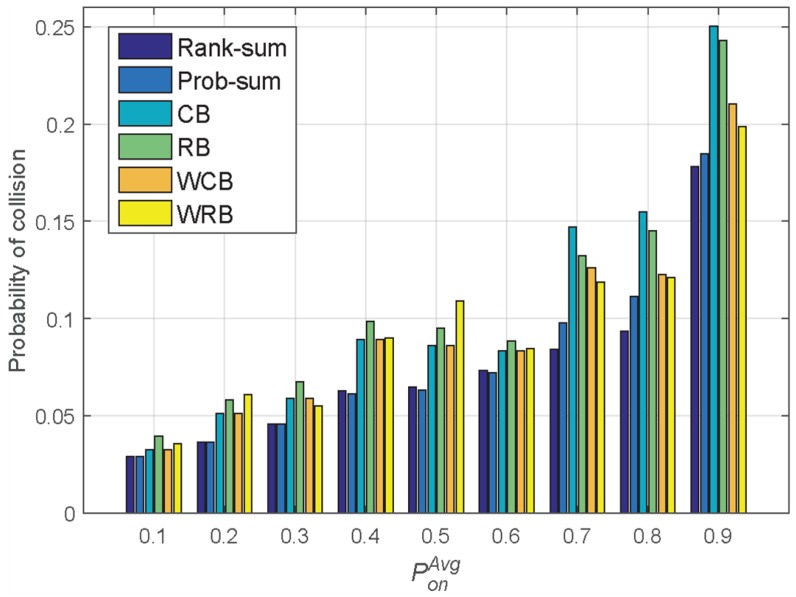
Comparison of collision probability for each sampling method according to changes in the traffic (reasoning period = 20 slots, number of samples = 20 slots, sample interval = 5 slots).

In [8], the relationship between the channel availability and the success in channel use was realized by conducting real-world simulation in a testbed. According to the testbed result in [8], it could be confirmed that the success in channel use was becoming below 40% when the channel availability was less than 10%. From the testbed result, there was a significant event that the success in channel use became abruptly degraded when the channel availability dropped at a certain point. Similarly, in our paper, the relationship between $P_{on}^{Avg}$ like the channel availability in [8] and the collision probability can be translated into the success in channel use in [8] observed in Figure 13, whose trend looks quite similar to that between the channel availability and the success in channel use in [8]. The only difference between the result in [8] and ours in Figure 13 is attributed to the domain, whether PU activity is considered in the temporal domain for the fixed channel or in the spectrum domain for the fixed time interval. Our result focuses on the temporal domain for a certain frequency channel, so that the collision probability is the number of time slots in which the PU and the SU coexist.

Figure 13 shows the results of collision probability depicted for the purpose of comparison and is tightly associated with the PU traffic model in Figure 12. Along with the simulation test, the CB, RB, WCB, and WRB sampling methods in the learning engine and the rank-sum and prob-sum reasoning methods were each set to have 20 samples, a reasoning period of 20 slots, and a sampling interval of five slots. In the simulation test results in Figure 13, when the PU traffic model’s channel occupancy probability value $P_{on}^{Avg}$ is low, there are many channels available because the occupancy probabilities are relatively low, and the collision probability becomes low for every sampling and reasoning method. Conversely, once $P_{on}^{Avg}$ gradually increases, the PU traffic becomes dense, and naturally, the probability of a collision increases due to the frequent change in the PU’s occupancy pattern.

With the uniform sampling method, it seems that the PU traffic model results in a relatively low collision probability for $P_{on}^{Avg}$ values of 0.1, 0.2, or 0.3. This implies that whenever the PU occupancy pattern is sparse, the uniform sampling method gives rise to candidate channels with high fidelity. For the other cases with the weighted sampling method, the collision probability is sustained at a low level compared to the uniform sampling case, even though the $P_{on}^{Avg}$ values are 0.7, 0.8, or 0.9. This means that the weighted sampling method is superior to the uniform sampling approach in the aspect of the resulting low level of the collision probability whenever there is a continuous PU occupancy pattern.

#### 4.2.2. Collision Probability Behavior According to Reasoning Period for a Fixed Number of Samples

**Figure 14:**
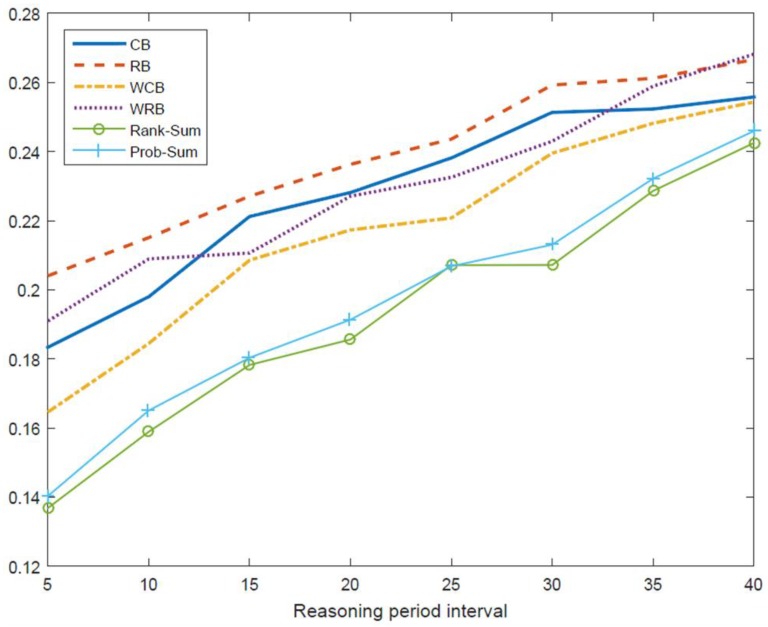
Comparison of collision probability according to reasoning period (number of samples = 20 slots, sample interval = 5 slots).

The simulation results in Figure 14 show the collision probability when the number of samples and the sampling interval are fixed at 20 samples and five slots, respectively, and the reasoning period is enforced to be changed from five to 40 slots. The reasoning period is a critical parameter, where the probability of PU occupancy is calculated and the available channels are determined. To adapt the dynamic PU activity, it is clear that the reasoning period should be adjusted with the help of acquiring current PU occupancy status. Assuming that the channel used for the next reasoning period is determined by conducting a series of the ordering and ranking method proposed in our paper, it can exploit the relationship between the reasoning period and the collision probability shown in Figure 14. Conceptually, for the sake of selecting an appropriate reasoning period, the result as in Figure 14 can be utilized as a referenced guideline subject to obeying the prescribed requirement such as the collision probability.

In Figure 14, it is evident that the PU collision probability increases as the reasoning period becomes longer for a fixed number of samples taken using the same sampling interval. This means that a highly reliable policy can be established if the reasoning period becomes as short as possible. If the required collision probability level is set to 25%, this implies that the reasoning period must be 25 slots or less.

#### 4.2.3. Collision Probability Behavior According to the Number of Samples for a Fixed Reasoning  Period

**Figure 15:**
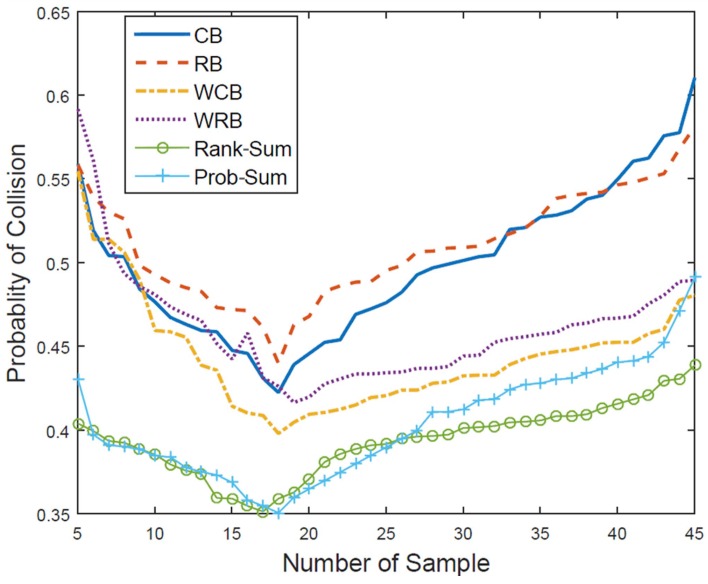
Comparison of collision probability according to the number of samples (reasoning period = 20, sample interval = 5).

In general, the collision probability between the primary user and the CR node user is directly related to the activity of PU’s channel occupancy. In other words, if the activity of PU’s channel occupation increases, the probability of collision is increased, as shown in Figure 15. Furthermore, it can also be stated that the probability of collision increases as the averaged occupancy probability per PU rises. Therefore, the relevant comparison of the collision probabilities as the indicator for the CR engine proposed in this paper is considered to be reasonable provided that the parameters for verifying the learning engine and the reasoning engine are consistent under the same environment of PU channel occupation. Note that in the field test of the XG project, the target success rate was confined to 60%, which reads 40% of the failure rate in channel use between the CR node and the primary user [8].

Surely, it is critical which value should be determined for the number of samples in order to estimate the value of the probability of PU occupancy accurately. However, due to the dynamic characteristic of PU occupancy, depending on how many samples are used, the collision probability could be altered. Thus, it could not be said that the longer depth of samples is always best for every situation. In other words, once the PU traffic pattern is apprehended by conducting histogram shape matching, referring to the result, an appropriate number of samples should be explored. Accordingly, Figure 15 delivers the meaning of this effect, which resulted from executing the exhaustive work, and it can be clarified that the number of samples can be determined by selecting the value at the point where the corresponding collision probability is the lowest. The simulation test results in Figure 15 show the collision probability when the reasoning period and the sampling interval are fixed to 20 slots and five slots, respectively, and the number of samples is changed from five to 45.

The simulation results in Figure 15 reveal that when the number of samples required to calculate the occupancy probability changes while the reasoning period remains the same, the lowest collision probability can be achieved by executing a proper reasoning process if the number of samples is in the range of 16–18. In uniform sampling methods, such as the CB and RB methods, the collision probability becomes rapidly enlarged as the number of samples becomes larger. Conversely, in the WCB and WRB methods, when the number of samples increases, there is not a noticeable effect on the performance of the candidate channel reasoning. Furthermore, if the number of samples is insufficiently small or unnecessarily large, the collision probability becomes high, so the reasoning capability decreases and the reliability is low. Specifically, for a long period of the sampling interval, it is not possible to respond quickly to rapid changes in the PU occupation state.

## 5. Conclusions

This paper proposes a novel cognitive engine platform structure comprising a cognition engine, learning engine, and reasoning engine. The proposed structure is suitable for future tactical cognitive sensor networks by enabling dynamic spectrum access in the presence of incumbent devices, namely the PU. To minimize collisions with the PU, a PU traffic model obeying a specific probability distribution is generated assuming the perfect spectrum sensing technology is operated under the environment reflecting various PU activity patterns. The performance of the learning engine conducting the estimation of the PU occupancy probability was confirmed via carrying out comparative studies employing either the currently-available or the proposed sampling methods. This paper also introduces a case-based reasoning engine to exploit candidate channels for a TCRN by taking into account the PU’s channel occupancy patterns. In the simulation, the CB, RB, WCB, and WRB sampling methods followed by rank-sum and prob-sum reasoning methods were applied to estimate the PU occupancy probability and to extract highly reliable candidate channels. Moreover, the collision probability and its behaviors were analyzed as performance indicators to verify the superiority of the proposed cognitive engine platform. Through the simulation results, we confirmed that the operation of the CR engine is possible through the learning and reasoning process about whether or not the primary user channel is occupied in the past time. Through this, it is possible to expand the research into the CR engine to perceive various cases encompassing the situation of the appearance of the jamming signal and rendezvous situation of the new CR node. In addition, instead of employing exhaustive search, a heuristic estimation of the learning and inference parameters proposed in this paper can be processed by applying optimization methods such as the genetic algorithm and Hungarian algorithm, resulting in reduced computational complexity. Moreover, besides the proposed temporal slotted process, to perform a more accurate case-based reasoning in support of the learning engine, the developed approach is preferable to configure the relevant parameters including available channels for TCRNs via conducting precise determination whether if the harmful collision happened both in the temporal and the spectral domain. Here, as a way to execute the spectrum domain approach, the Monte Carlo simulation is suitable for exploiting the insightful result while obeying the prescribed requirements such as I/N and SINR thresholds with regard to the spatial distribution of the PU and the TCRNs.

## Figures and Tables

**Table 1:** IEEE P1900.5 standard’s cognitive radio engine components [17].

Components	Main Functions
PMP (policy management point)	Provide and manage policy information related to radio regulations and provide new policy data. This corresponds to frequency managers, system administrators, and operators
SSRC (system strategy reasoning capability)	Opportunistically check for access opportunities using REM (radio environment map) data and request information on whether or not transmission to PCR is possible
PCR (policy conformance reasoner)	Compare SSRC requests and existing policies transmitted from the PMP, perform the reasoning process, verify the suitability of changed policies, and approve or reject SSRC requests
PE (policy enforcer)	Execute communications service based on verified policy information transmitted from the PCR or SSRC

**Table 2:** Main features of uniform sampling and weighted sampling methods.

Sampling Methods	Main Features
Uniform Sampling	• (CB) Divides the time slots to be sampled at a fixed time slot in the sampling interval
	• (RB) Divides the time slots to be sampled at a random time slot in the sampling interval slots of random size and position
Weighted Sampling	• (WCB) Assigns exponentially-decaying weights from the present to the past unit time slots sampled by CB.
	• (WRB) Assigns exponentially-decaying weights from the present to the past unit time slots sampled by RB

**Table 3:** Examples of the reference PU traffic models.

PU Traffic Model	1	2	3	…	9
Mean (μ)	0.1	0.2	0.3	…	0.9
Variance ($\mu_{2}$)	0.025	0.052	0.069	…	0.025
Skewness ($\gamma_{1}$)	2.888	1.569	0.850	…	−2.776
Kurtosis ($\gamma_{2}$)	9.540	1.792	0.308	…	9.505

**Table 4:** Calculated occupancy probability and channel ranking.

	CB		RB		WCB		WRB		Rank-Sum		Prob-Sum	
Rank	Ch.	Prob.	Ch.	Prob.	Ch.	Prob.	Ch.	Prob.	Ch.	Sum	Ch.	Sum
1	Ch. 2	0.58%	Ch. 1	5.43%	Ch. 2	5.54%	Ch. 2	3.26%	Ch. 2	6	Ch. 2	28.23%
2	Ch. 1	10.61%	Ch. 3	16.48%	Ch. 3	5.98%	Ch. 1	8.31%	Ch. 3	9	Ch. 1	45.64%
3	Ch. 3	11.84%	Ch. 2	18.85%	Ch. 4	11.27%	Ch. 3	15.08%	Ch. 1	11	Ch. 3	48.15%
4	Ch. 4	12.95%	Ch. 4	20.36%	Ch. 6	15.33%	Ch. 6	20.58%	Ch. 4	16	Ch. 4	66.44%
5	Ch. 5	19.06%	Ch. 5	22.12%	Ch. 1	20.06%	Ch. 4	21.86%	Ch. 6	20	Ch. 5	88.51%
6	Ch. 6	31.95%	Ch. 6	24.72%	Ch. 5	25.14%	Ch. 5	22.19%	Ch. 5	22	Ch. 6	92.58%

**Table 5:** Simulation test parameter types and setting values.

Parameter Type	Setting Value
Overall number of channels, *N*	500
Overall time slot interval, *T*	2700 slots
PU traffic model’s unoccupied channel probability mean value, $P_{on}^{Avg}$	0.1, 0.2, …, 0.9
Number of PU traffic models, *I*	9
Time slot interval for each traffic model	300 slots
